# An innovative fast track solution for food bolus impaction due to Jackhammer esophagus in an emergency department: the “Nitro-Push Blind Technique” case report

**DOI:** 10.1186/s12876-016-0511-7

**Published:** 2016-08-18

**Authors:** Luigi Marano, Alessandro Cecchi, Federica Chiodo, Francesco Gullo, Pasquale Fiorillo, Luca Roncetti, Mattia Longaroni, Gianluca Proietti Silvestri, Silvano Lolli, Giorgio Nicolic, Alberto Patriti

**Affiliations:** 1 Unit of Robotic Surgery, “San Matteo degli Infermi Hospital” – ASL Umbria 2, 06049 Spoleto (PG), Italy; 2 Department of Emergency Medicine and Surgery, “San Matteo degli Infermi Hospital” – ASL Umbria 2, Via Loreto 3, 06049 Spoleto (PG), Italy

**Keywords:** Esophageal food bolus impaction, Jackhammer esophagus, Nutcracker esophagus, Case report

## Abstract

**Background:**

In the medical literature are described only few clinical cases of esophageal food bolus impaction due to esophageal motility disorders. Moreover, the management of this condition is highly variable with no evidence in the literature to strongly support a clear defined intervention.

**Case presentation:**

In this paper we describe for the first time a case of 53-year-old male with food bolus impaction due to Jackhammer esophagus referred to emergency department. On the basis of the known esophageal past medical history as well as the absence of bones in the bolus, the patient was submitted to a new conservative treatment, the “Nitro-Push Blind Technique”.

**Conclusions:**

The new technique performed with naso-gastric tube thrust after nitrates medication in definite clinical case supported by known functional disease, represents a safe and successful method, with short observational period to minimize exposure to potential morbidity and reduce the inpatient stay in emergency department. It should be recommended, once validated in a larger cohort, as the initial treatment of choice in the selected patients with food boneless bolus impaction in the emergency settings. Indeed, this management provides only minimal deviation from the current practice and is hence technically easy to learn and perform.

## Background

Esophageal food bolus impaction is a multidisciplinary common emergency that frequently requires presentation to the Emergency Department for diagnosis and management [1]. The estimated annual incidence rate is 13 per 100.000 persons ranking third after upper and lower gastrointestinal bleeding [2]. The most common presentation of food bolus impaction in the Western countries are the “steakhouse syndrome”, so called because the meats without bones represent the most frequently impacted foods [3]. As hollow muscular tube with virtual lumen, the esophagus results in a common site of food impaction. Moreover, several abnormalities of esophageal mucosal and neuromuscular layers as well as underlying diseases, including edentoulism, eosinophilic esophagitis and esophageal stenosis (peptic esophageal stricture or Schatzki’s ring), facilitate foreign body impactions [3, 4]. More infrequently, esophageal motility disorders including diffuse esophageal spasm, nutcracker esophagus and other esophageal hyperthensive dysmotilities may represent a cause of food bolus impaction [5]. The first description of hypertensive motility disorders was realized with conventional manometry: Castell DO et al. [6] defined “nutcracker esophagus” as a condition in which patients with unexplained chest pain and/or dysphagia exhibit peristaltic contractions in the distal esophagus with mean amplitudes exceeding normal values by more than two standard deviation; several years later, Spechler SJ et al. [7] defined “hypertensive peristalsis” as a mean distal esophageal peristaltic wave amplitude, measured as the average amplitude of 10 swallows at two recording sites positioned 3 and 8 cm above the lower esophageal sphincter, higher than 180 mmHg. However, more recently, in the era of Clouse plot and high-resolution manometry (HRM), a new metric, the Distal Contractile Integral (DCI), is used to assess contractile vigor, with the consequence of newly introduced term to describe one of the major motility disorders: jackhammer (or hypercontractile) esophagus, defined as a condition in which it is recognizable more than 20 % of swallows with a DCI ≥8000 mmHg · s · cm and normal latency, instead of nutcracker (or hypertensive) esophagus, considered variations of normal findings and retained of unclear clinical significance [8].

To the best of our knowledge only few clinical cases of food bolus impaction due to esophageal motility disorders are described in the medical literature [5, 9], and, as a consequence, the management of this condition is highly variable with no evidence in the literature to strongly support a clear defined intervention.

In this paper we describe for the first time a case of food bolus impaction due to Jackhammer esophagus treated in an emergency department, with discussion of the cornerstones in management and description of an innovative as well as conservative fast track solution, the so called “Nitro-Push Blind Technique”.

## Case presentation

A 53-year-old Caucasian sightseer man, with past medical history significant for non-specific hypertensive motility disorders of the esophagus with incomplete or spontaneously resolved episodes of esophageal obstruction (the last one three months ago, with a global annual frequence of 3–4 episodes, as referred), was eating his grilled sirloin steak with trouffle dinner at a restaurant in Spoleto, italian city extremely renowned for “The Festival of Two Worlds”, when he felt swallowing difficulty with stoppage of food passage. He is accompanied to the emergency department of district general hospital by his friends given the persistence of symptoms 15 min after the ingestion of boneless meat. The patient had no history of other specific diseases in the past and no abnormal family or medication history of note. He denied prior weight loss, halitosis, ingestions of caustic substances, excessive alcohol consumption, hematemesis and melena. Physical examination, including firstly an airway evaluation and ventilatory status assessment, resulted unremarkable. On the basis of the known esophageal past medical history as well as the absence of bones in the bolus, the patient was submitted to a new conservative treatment, the “Nitro-Push Blind Technique”: after suitable preparation and monitoring of blood pressure, two sprays (600 mcg) of nitroglycerine (Natispray®, Teofarma srl, Valle Salimbene, PV, Italy) were administered under the tongue. Five minutes later, a 28 Fr naso-gastric tube (Argyle™ Lavacuator Tube, Covidien, Mansfield, MA, USA) was carefully and blindly inserted into esophageal lumen until resistance to progression and, during the air insufflation at this level through the tube’s service channel, gentle pressure was applied to the food bolus by means of catheter’s tip to facilitate disimpaction. Sudden symptoms relief and copious belch indicated the resolution of obstruction obviating the need for endoscopic approach. Patient was carefully discharged after 30 min from admission without complications, with a written plan of home care advising the patient to go to urgent care in case of any symptoms as well as the recommendation to contact a gastroenterologist to define the diagnosis. At this aiming, after 4 days, the patient underwent esophageal HRM, performed with a 4 mm outer diameter 24-channel water-perfused system (Gastro Explorer HR®, EB Neuro Spa, Firenze, Italy). Esophageal pressure topography data was analyzed using Mano.Net® (EB Neuro Spa, Firenze, Italy) analysis software. The acquired HRM datasets were reviewed according to the current Chicago classification [8] and the hypercontractile pattern with regular lower esophageal relaxation, normal wave front velocity but hypertensive, repetitive contractions (>300 mmHg), and a distal contractile integral >10,000 mmHg · s · cm were consistent with the diagnosis of Jackhammer esophagus.

## Conclusions

Esophageal food bolus impaction represents a common problem in emergency clinical practice involving several medical specialities (emergency medicine, gastroenterology, otolaryngology, general and thoracic surgery). This interdisciplinarity as well as the plurality of underlying esophageal diseases generate an high heterogeneity in its management, ranging from observational therapy to surgical treatment [10]. Furthermore, “steakhouse syndrome” has infrequently been associated with esophageal motility disorders and only four clinical cases addressing the optimal management are described in the medical literature [5, 9], with the estimated actual incidence of dysmotility disorders associated with bolus impaction of about 14 % [10]. The true epidemiology of food bolus impaction due to esophageal motility disorders has been unclear for many years because of lack of knowledge of these dysmotilities resulting in an underestimation. In this paper we describe for the first time a case of food boneless bolus impaction due to Jackhammer esophagus, an extreme phenotype of esophageal hypercontractility [8].

Although more than half of food bolus impaction will resolve spontaneously after a short observational period without any serious consequences, in 10–20 % of cases a conservative or surgical management is required [11, 12]. Several studies have been conducted on pharmacological and non-pharmacological agents aiming at investigation of efficacy in food bolus dislogdgment by means of gas-forming, enzymatic or spasmolytic properties, however the efficacy of these molecules are still unclear [13]. Pharmacological agents with enzymatic activity such as papain, trypsin and chymotrypsin are no longer recommended due to high risk of esophageal perforation and hypernatremia. In the same way, the administration of hyoscine butylbromide (Buscopan), glucagone injection and diazepam resulted in no significant difference in the disimpaction rate between the treated patients when compared with controls [13]. Better results were obtained by non-pharmacological effeverscent agents (fizzy drinks), seeming to be effective at successfully resolution of esophageal impaction in a number of cases. However the level of evidence of these studies is too low to provide strong conclusions. Since our patient reported a past medical history suggestive of esophageal hypertensive motility disorders we decided to administer two sprays (600 mcg) of nitroglycerine under the tongue, hypothesizing that acute esophageal spasm could be the causative factor for obstruction. Even though the nitrates have not been used so far to treat food impaction in the emergency settings, they are strongly recommended for the treatment of esophageal hypertensive motility disorders [13]. These agents are proven to reduce the pressure of lower esophageal sphincter as well as the peristaltic contraction pattern improving the chest disconfort. Moreover, taking advantage of the smooth muscles relaxation, a gentle pressure blindly applied to the bolus by means of naso-gastric tube facilitated successfully the disimpaction, avoiding that the prolonged pressure of bolus on the esophageal wall can result in ischemic injury with perforation. The current treatments recommended by American Society for Gastrointestinal Endoscopy (ASGE) include endoscopic food extraction and, only in some cases, the advancement of the bolus into the stomach under endoscopic guide [14]. Nonetheless, it is still debated what is the more preferable bolus management. Several authors [14] consider unsafe the push technique due to risk of esophageal wall damage, especially in case of suspected organic stenosis, unknown esophageal anatomy or past medical history consistent for dysphagia. However, Vicari JJ et al. [15] and Longstreth GF et al. [2], in two large series for a total of 375 patients, reported the application of gentle pressure on the bolus without any complications such as perforation or bleeding, considering the bolus extraction as second choice procedure for more resistant boluses. In our definite clinical case supported by known functional disease, the push technique performed with naso-gastric tube after nitrates medication was a safe and successful method, with short observational period to minimize exposure to potential morbidity and reduce the inpatient stay. It may be recommended, once validated in a larger cohort, as the initial treatment of choice in the selected patients with food boneless bolus impaction in the emergency settings. Indeed, this management provides only minimal deviation from the current practice and is hence technically easy to learn and perform.

## Abbreviations

ASGE: American Society for Gastrointestinal Endoscopy
DCI: Distal Contractile Integral
HRM: High resolution manometry

## References

[1] Price T, Jones SE, Montgomery PQ (2007). Is current UK management of oesophageal food bolus obstruction evidence based? an e-mail survey and literature review. Eur Arch Otorhinolaryngol.

[2] Longstreth GF, Longstreth KJ, Yao JF (2001). Esophageal food impaction: epidemiology and therapy. A retrospective, observational study. Gastrointest Endosc.

[3] Stadler J, Holscher AH, Feussner H (1989). The “Steakhouse syndrome” - Primary and definitive diagnosis and therapy. Surg Endosc.

[4] Sperry SL, Crockett SD, Miller CB (2011). Esophageal foreign-body impactions: epidemiology, time trends, and the impact of the increasing prevalence of eosinophilic esophagitis. Gastrointest Endosc.

[5] Chae HS, Lee TK, Kim YW (2002). Two cases of steakhouse syndrome associated with nutcracker esophagus. Dis Esophagus.

[6] Benjamin SB, Gerhardt DC, Castell DO (1979). High amplitude, peristaltic esophageal contractions associated with chest pain and/or dysphagia. Gastroenterology.

[7] Spechler SJ, Castell DO (2001). Classification of oesophageal motility abnormalities. Gut.

[8] Kahrilas PJ, Bredenoord AJ, Fox M (2015). The Chicago Classification of esophageal motility disorders, v3.0. Neurogastroenterol Motil.

[9] Breumelhof R, Van Wijk HJ, Van Es CD (1990). Food impaction in nutcracker esophagus. Dig Dis Sci.

[10] Reddy VM, Bennett W, Burrows SA (2011). Recurrence of food bolus impaction of the oesophagus: a retrospective observational study. Int J Surg.

[11] Basavaraj S, Penumetcha KR, Cable HR (2005). Buscopan in oesophageal food bolus: is it really effective?. Eur Arch Otorhinolaryngol.

[12] Sodeman TC, Harewood GC, Baron TH (2004). Assessment of the predictors of response to glucagon in the setting of acute esophageal food bolus impaction. Dysphagia.

[13] Khayyat YM (2013). Pharmacological management of esophageal food bolus impaction. Emerg Med Int.

[14] Ikenberry SO, Jue TL, ASGE Standards of Practice Committee (2011). Management of ingested foreign bodies and food impactions. Gastrointest Endosc.

[15] Vicari JJ, Johanson JF, Frakes JT (2001). Outcomes of acute esophageal food impaction: success of the push technique. Gastrointest Endosc.

